# Supplementary material for: Effect of rotenone-induced stress on physiologically active substances in adult Aphis glycines
Source: PLoS One. 2020 Jun 4;15(6):e0234137. doi: 10.1371/journal.pone.0234137 (PMC7272001; doi:10.1371/journal.pone.0234137)
Supplement: Attachment — Submitted filename: PONE-D-19-35139_reviewer.pdf [file pone.0234137.s002.pdf]

PLOS ONE

# Effect of rotenone-induced stress on physiologically active substances in adult Aphis glycines

--Manuscript Draft--

| | |
|---|---|
| **Manuscript Number:** | PONE-D-19-35139 |
| **Article Type:** | Research Article |
| **Full Title:** | Effect of rotenone-induced stress on physiologically active substances in adult Aphis glycines |
| **Short Title:** | Effect of LC50 rotenone on Aphis glycines |
| **Corresponding Author:** | Kuijun Zhao<br>Northeast Agricultural University<br>Harbin, CHINA |
| **Keywords:** | |
| **Abstract:** | The aim of this study was to determine the effect of rotenone stress on Aphis glycines populations in different habitats in Northeast China. The changes in kinase expression activity of endogenous substances (proteins, total sugars, trehalose, cholesterol, and free amino acids), detoxifying enzymes (cytochrome P450 and glutathione-S-transferase), and metabolic enzymes (proteases and phosphofructokinases) in specimens from field populations A and B and a laboratory population were compared before and after stress with rotenone at median lethal concentration (LC50) and their response mechanisms were analyzed. Following a 24-h treatment with rotenone at LC50, the LC50 levels for the specimens from the three populations were 4.3859, 4.6088, and 4.0305 mg/mL, respectively. The degree of changes in the kinase expression activity of endogenous substances also differed, which indicated a difference in the response of A. glycines from varying habitats to LC50 rotenone stress. The content of endogenous substances, detoxifying enzymes, and metabolic enzymes, except for free amino acids, changed significantly in all populations treated with rotenone at LC50 compared with that in the control ($P < 0.05$). The decrease in protein and trehalose content and the obstruction of cholesterol transportation due to decreased feeding were one of the causes of A. glycines death after rotenone treatment. A. glycines resistance to rotenone may be related to cytochrome P450 expression. |
| **Order of Authors:** | Lanlan Han |
| | Litong Gao |
| | Kuijun Zhao |
| | Wenlin Zhang |
| | Juan Chen |
| | Jianfei Xiao |
| | Aonan Zhang |
| | Ziru Hao |
| | Zhenghao Shi |
| | Lin Zhu |
| **Additional Information:** | |
| **Question** | **Response** |
| **Financial Disclosure**<br><br>Enter a financial disclosure statement that describes the sources of funding for the | This work was supported by Special Fund for Construction of Modern Agricultural Industry Technology System (grant number CARS-04); Heilongjiang Science Foundation Project (grant number C2018011). |

Powered by Editorial Manager® and ProduXion Manager® from Aries Systems Corporation

| | |
|---|---|
| work included in this submission. Review the submission guidelines for detailed requirements. View published research articles from *PLOS ONE* for specific examples.<br><br>This statement is required for submission and **will appear in the published article** if the submission is accepted. Please make sure it is accurate.<br><br>**Unfunded studies**<br>Enter: *The author(s) received no specific funding for this work.*<br><br>**Funded studies**<br>Enter a statement with the following details:<br>• Initials of the authors who received each award<br>• Grant numbers awarded to each author<br>• The full name of each funder<br>• URL of each funder website<br>• Did the sponsors or funders play any role in the study design, data collection and analysis, decision to publish, or preparation of the manuscript?<br>• **NO** - Include this sentence at the end of your statement: *The funders had no role in study design, data collection and analysis, decision to publish, or preparation of the manuscript.*<br>• **YES** - Specify the role(s) played.<br><br>* typeset | |
| **Competing Interests**<br><br>Use the instructions below to enter a competing interest statement for this submission. On behalf of all authors, disclose any competing interests that could be perceived to bias this work—acknowledging all financial support and any other relevant financial or non-financial competing interests.<br><br>This statement **will appear in the published article** if the submission is accepted. Please make sure it is accurate. View published research articles from *PLOS ONE* for specific examples. | The authors have declared that no competing interests exist. |

| | |
|---|---|
| **NO authors have competing interests**<br><br>Enter: *The authors have declared that no competing interests exist.*<br><br>**Authors with competing interests**<br><br>Enter competing interest details beginning with this statement:<br><br>*I have read the journal's policy and the authors of this manuscript have the following competing interests: [insert competing interests here]*<br><br>* typeset | |
| **Ethics Statement**<br><br>Enter an ethics statement for this submission. This statement is required if the study involved:<br><br>• Human participants<br>• Human specimens or tissue<br>• Vertebrate animals or cephalopods<br>• Vertebrate embryos or tissues<br>• Field research<br><br>Write "N/A" if the submission does not require an ethics statement.<br><br>General guidance is provided below. Consult the submission guidelines for detailed instructions. **Make sure that all information entered here is included in the Methods section of the manuscript.** | N/A |

Powered by Editorial Manager® and ProduXion Manager® from Aries Systems Corporation

| | |
|---|---|
| **Format for specific study types**<br><br>**Human Subject Research (involving human participants and/or tissue)**<br>• Give the name of the institutional review board or ethics committee that approved the study<br>• Include the approval number and/or a statement indicating approval of this research<br>• Indicate the form of consent obtained (written/oral) or the reason that consent was not obtained (e.g. the data were analyzed anonymously)<br><br>**Animal Research (involving vertebrate animals, embryos or tissues)**<br>• Provide the name of the Institutional Animal Care and Use Committee (IACUC) or other relevant ethics board that reviewed the study protocol, and indicate whether they approved this research or granted a formal waiver of ethical approval<br>• Include an approval number if one was obtained<br>• If the study involved *non-human primates*, add *additional details* about animal welfare and steps taken to ameliorate suffering<br>• If anesthesia, euthanasia, or any kind of animal sacrifice is part of the study, include briefly which substances and/or methods were applied<br><br>**Field Research**<br><br>Include the following details if this study involves the collection of plant, animal, or other materials from a natural setting:<br>• Field permit number<br>• Name of the institution or relevant body that granted permission | |
| **Data Availability**<br><br>Authors are required to make all data underlying the findings described fully available, without restriction, and from the time of publication. PLOS allows rare exceptions to address legal and ethical concerns. See the PLOS Data Policy and FAQ for detailed information. | Yes - all data are fully available without restriction |

Powered by Editorial Manager® and ProduXion Manager® from Aries Systems Corporation

| | |
|---|---|
| A Data Availability Statement describing where the data can be found is required at submission. Your answers to this question constitute the Data Availability Statement and **will be published in the article**, if accepted.<br><br>**Important:** Stating 'data available on request from the author' is not sufficient. If your data are only available upon request, select 'No' for the first question and explain your exceptional situation in the text box.<br><br>Do the authors confirm that all data underlying the findings described in their manuscript are fully available without restriction? | |
| **Describe where the data may be found in full sentences. If you are copying our sample text, replace any instances of XXX with the appropriate details.**<br><br>• If the data are **held or will be held in a public repository**, include URLs, accession numbers or DOIs. If this information will only be available after acceptance, indicate this by ticking the box below. For example: *All XXX files are available from the XXX database (accession number(s) XXX, XXX.*).<br>• If the data are all contained **within the manuscript and/or Supporting Information files**, enter the following: *All relevant data are within the manuscript and its Supporting Information files.*<br>• If neither of these applies but you are able to provide **details of access elsewhere**, with or without limitations, please do so. For example:<br><br>*Data cannot be shared publicly because of [XXX]. Data are available from the XXX Institutional Data Access / Ethics Committee (contact via XXX) for researchers who meet the criteria for access to confidential data.*<br><br>*The data underlying the results presented in the study are available from (include the name of the third party* | All relevant data are within the manuscript and its Supporting Information files. |

| | |
|---|---|
| *and contact information or URL).*<br>• This text is appropriate if the data are owned by a third party and authors do not have permission to share the data.<br><br>* typeset | |
| Additional data availability information: | |

Powered by Editorial Manager® and ProduXion Manager® from Aries Systems Corporation

Manuscript Click here to access/download;Manuscript;Manuscript.doc



# Effect of rotenone-induced stress on physiologically active substances in adult *Aphis glycines*

Han Lanlan¶ [1], Gao Litong ¶ [1], Zhao Kuijun* [1], Zhang Wenlin[1], Chen Juan[1], Xiao Jianfei[1], Zhang Aonan[1], Hao Ziru[1], Shi Zhenghao[1], Zhu Lin[1]

[1] Agricultural Insect and Pest Control Task Group, College of Agriculture, Northeast Agricultural University, Harbin, Heilongjiang, China

* Corresponding author

E-mail: kjzhao@neau.edu.cn (ZK)

¶ First author: These authors contributed equally to this work

**Short title**: Effect of $LC_{50}$ rotenone on *Aphis glycines*

# Abstract

The aim of this study was to determine the effect of rotenone stress on *Aphis glycines* populations in different habitats in Northeast China. The changes in kinase expression activity of endogenous substances (proteins, total sugars, trehalose, cholesterol, and free amino acids), detoxifying enzymes ~~(cytochrome P450 and glutathione-S-transferase), and metabolic enzymes (proteases and phosphofructokinases)~~ in specimens from field populations A and B and a laboratory population were compared before and after stress with rotenone at median lethal concentration ($LC_{50}$) and their response mechanisms were analyzed. Following a 24-h treatment with rotenone at $LC_{50}$, the $LC_{50}$ levels for the specimens from the three populations were ~~4.3859~~, ~~4.6088~~, and ~~4.0305~~ mg/mL, respectively. The degree of changes in the kinase expression activity of endogenous substances also differed, which indicated a difference in the response of *A. glycines* from varying habitats to $LC_{50}$ rotenone stress. The content of endogenous substances, detoxifying enzymes, and metabolic enzymes, except for free amino acids, changed significantly in all populations treated with rotenone at $LC_{50}$ compared with that in the control ($P < 0.05$). The decrease in protein and trehalose content and the obstruction of cholesterol transportation due to decreased feeding were one of the causes of *A. glycines* death after rotenone treatment. *A. glycines* resistance to rotenone may be related to cytochrome P450 expression.

**Keywords:** *Aphis glycines*; rotenone; median lethal concentration ~~($LC_{50}$)~~; physiologically active substance; cropping pattern

# Introduction

*Aphis glycines* (Hemiptera: Aphididae~~; *Aphis*~~) is one of the main pests on soybeans and harms soybean plants by feeding on the leaves and causing undesirable effects such as soybean leaf curling and plant dwarfing [1], which in turn leads to a series of economic problems such as decreased soybean yield and reduced quality [2]. Currently, the prevention and control of *A. glycines* is primarily based on chemical methods, but the abuse of chemical pesticides has not only caused certain damage to the environment, but also resulted in pesticide-resistant *A. glycines* [3]. Around 2004, in Mudanjiang (Heilongjiang Province, China), although the dosage of dimethoate used to control *A. glycines* has almost doubled, its efficacy is still declining [4]. Therefore, utilizing integrated pest management systems, making scientific and rational use of chemical pesticides, reducing damage to farmland ecosystems, and controlling harmful organisms below the allowable level of economic damage are the focus of recent studies [5].

The outbreak and damage caused by *A. glycines* is due to the interaction of multiple factors, including aphids, natural enemies, soybeans, and environmental factors. The initial period of *A. glycines* infection in the field is generally in the middle of June, and the peak slightly varies with year, generally in early July or end of July. The abundance of *A. glycines* gradually decreases after reaching the peak, and it disappears in the fields by the end of August to the beginning of September. The abundance of natural enemies gradually peaks with the increase in the abundance of *A. glycines*. The natural enemies occur approximately 15 days after the occurrence of *A. glycines*, and they disappear approximately 20 days before the disappearance of soybean aphids. The population of natural enemies peaks at 5–10 days earlier than that of *A.*

*glycines*. Therefore, prevention and control of aphids can effectively control their spread to the whole field before the peak of aphid population [6]. Currently, several studies have tried to use biodiversity to control pests. Some studies have shown that intercropping and adjacent cropping patterns have a regulatory effect on *A. glycines* populations, and this approach, along with the natural enemies of *A. glycines*, can inhibit the growth rate of *A. glycines*. When soybean and corn were intercropped at ratios of 8:2 and 8:8, the first peak number of *A. glycines* was lower than that in clear soybean fields [7]. A comprehensive investigation showed that among the soybean and early-maturing potato fields, intercropped at a ratio of 2:2, 6:6, 8:8, 16:16, and 32:32, the field intercropped at a ratio of 8:8 offered the best control against *A. glycines* with a reducing effect on its population density [8]. When potato-soybean and corn-soybean neighbor cropping patterns were adopted, the peak number of *A. glycines* was lower than that in control fields [9]. These studies prove that the use of biodiversity to control pests is an effective method of pest control.

Rotenone, extracted from the roots of leguminous plants, is a broad-spectrum plant insecticide. It causes stomach and contact toxicity, and acts as an antifeedant and a fumigant with control effects on the pests of 137 families in 15 orders [10, 11]. Rotenone can inhibit cell respiration; it is an electron transfer inhibitor that blocks electron transfer from nicotinamide adenine dinucleotide to coenzyme Q. Rotenone is a natural compound that can degrade fast with low toxicity. It is a pesticide that can meet the needs of an ecologically aware civilization [10].

The processes of growth, development, metamorphosis, and reproduction of insects are inseparable from the synthesis, decomposition, and transformation of proteins, lipids,

carbohydrates, and other substances in the insect body. The content of metabolic substances affects the growth and development of insects to a certain extent [12], thus reflecting the insect's ability to adapt to the environment. By measuring the changes in the content of metabolic substances in insects under the influence of external factors, it is possible to explore the internal relationships between various substances in insects.

In this study, we compared and analyzed the variable differences and trends in the protein, total sugar, trehalose, cholesterol, and free amino acid (FAA) content as well as protease, glutathione-S-transferase (GST), cytochrome P450 (CYP450), and phosphofructokinase (PFK) activities in adult *A. glycines* populations from three habitats under $LC_{50}$ rotenone stress to find a more efficient method to comprehensively control *A. glycines* and provide a theoretical basis for the effects of rotenone on this insect species.

# Materials and Methods

## Insect sources

*A. glycines* adults were collected in the Xiangyang farm, Northeast Agricultural University Harbin, Heilongjiang, China. *A. glycines* individuals collected in a corn and soybean neighbor cropping field were used as field population A and those collected in a potato and soybean neighbor cropping field were used as field population B. The adults of *A. glycines* for the laboratory population were collected from an artificial climate chamber (with ambient temperature: 24℃; photoperiod 16L:8D; and relative humidity: 60% ± 5%) in the laboratory, and were cultured continuously for more than 3 years.

## Determination of the effect of 24 h $LC_{50}$ rotenone treatment

## on *A. glycines*

We spread 1% agar medium in a 6-cm diameter plastic Petri dish and allowed it to solidify. We prepared 10 mL rotenone microemulsion formulations at concentrations of 16, 8, 4, 2, 1, and 0.5 mg/mL in 25 mL beakers. Fresh soybean leaves (approximately 1.5 $cm^2$ per piece) were immersed in each of the five prepared solutions for approximately 2 s, using clear water for the control group. The pieces were removed, pasted on the prepared medium, and labeled. Only one prepared leaf was pasted on each medium. The leaf pieces were allowed to dry. An adult *A. glycines* was placed on each leaf in each Petri dish. Three repetitions were set for each concentration, and each repetition had 20 *A. glycines* adults, four on each soybean leaf piece, that is five Petri dishes in each repetition.

After 24 h, the specimens were observed under a dissecting microscope as follows: the specimens were gently touched with a writing brush and observed for movement. If the specimen moved within 3–5 s, it was recorded as alive; if not, it was touched again and was recorded as alive if it moved. If it still did not move, it was recorded as dead. This procedure was repeated with each insect. The effect of the 24-h $LC_{50}$ rotenone treatment was calculated from the obtained death rate.

## Determination of metabolic substance content in *A. glycines*

*A. glycines* adults were stressed using the same leaf dipping method, as explained previously, with rotenone $LC_{50}$ for 24 h. After the stress treatment, live *A. glycines* of similar size were collected from the treatment and control groups, and 10 insects were placed in a 1.5-mL EP tube, cooled in liquid nitrogen, and refrigerated at -80°C for future use. Seventy *A. glycines* adults of

similar size were treated in each experiment. Thirty live insects were collected 24 h later, and the experiment was repeated several times until all physiological indicators were measured. These collected *A. glycines* were placed on ice blocks, and after adding 300 μL of PBS buffer, they were uniformly ground and mixed. The tissue homogenate was then centrifuged at 8000 × *g* for 10 min. The supernatant was transferred into a new EP tube and the content of physiologically active substances in *A. glycines* was determined using biochemical kits. The kits for protein, total sugar, trehalose, cholesterol, PFK, and GST were purchased from China Beijing Solarbio Science & Technology Co., Ltd. The FAA, CYP450, and protease kits were purchased from China Jiangsu Meibiao Biotechnology Co., Ltd.

## Data analysis

Statistical analysis software SPSS23.0 was used for data analyses. Independent sample *t*-test was adopted to compare the significant difference in physiologically active substances before and after chemical treatment. By using analysis of variance (ANOVA) combined with the least significant difference (LSD) method, multiple comparisons were made to analyze significant differences in physiologically active substances among the three populations, and the level of significance was $P = 0.05$.

# Results

## Determination of the virulence of rotenone against *A. glycines*

As shown in Table 1, the highest $LC_{50}$ value of rotenone in *A. glycines* tissues was from field population B at 4[illegible]8 mg/mL, followed by field population A at 4.3859 mg/mL, and the least was from the laboratory population at 4.0305 mg/mL.

**Table 1. Determination of virulence of rotenone against *A. glycines* from field population A, field population B, and laboratory population.**

| Testing population | $LC_{50}$ value (mg/mL) | Virulence regression equation | Correlation coefficient |
|---|---|---|---|
| Field population A | 4.3[illegible]（3.9423–4.8874） | Y = 3.9151 + 1.6888x | 0.9963 |
| Field population B | 4.6088（3.2897–6.4568） | Y = 3.8905 + 1.6720x | 0.9657 |
| Laboratory population | 4.0305（2.5626–6.3393） | Y = 4.0696 + 1.5369x | 0.9366 |

## Influence of rotenone L~~iiiiiiii~~$C_{50}$ stress on the content of protein metabolism-related substances in *A. glycines* adults of three populations

After being stressed with rotenone at $LC_{50}$, the content of protein decreased significantly ($F$ = 4.088, df = 4, $p$ = 0.003; $F$ = 10.469, df = 2.107, $p$ = 0.024; $F$ = 1.439, df = 4, $p$ = 0.017) in field populatio[illegible] field population B, and the laboratory population, with a decrease of 28.4%, 15.0%, and 20.3%, respectively. The activity of protease increased significantly ($F$ = 6.031, df = 4, $p$ = 0.033; $F$ = 0.437, df = 4, $p$ = 0.009; $F$ = 2.578, df = 4, $p < 0.0001$) in the three populations, with an increase of 26.5%, 41.3%, and 92.1%, respectively, whereas the content of FAA increased in all three populations, but it was not significant ($F$ = 3.906, df = 4, $p$ = 0.072; $F$ = 0.331, df = 4, $p$ = 0.268; $F$ = 7.180, df = 4, $p$ = 0.189), with an increase of 16.5%, 9.48%, and 19.8%, respectively (Fig 1).

**Fig 1. Influence of $LC_{50}$ rotenone on protein metabolism in different populations of *A.***

***glycines.***

The influence of $LC_{50}$ rotenone on protein content (A), protease content (B), and FAA content (C) in different populations of *A. glycines*. Different stripes in bars represent different treatments. * indicates that there was a significant difference ($p < 0.05$) between the chemical treatment group and control check group for the adults of *A. glycines* of the same population. Bar heights represent the sample mean and error bars are the standard error of the means.

## Influence of rotenone $LC_{50}$ stress on the content of sugar metabolism-related substances in *A. glycines* adults of three populations

After being stressed with rotenone at $LC_{50}$, the content of total sugar decreased in all three populations, and it was significant (F = 10.302, df = 2.134, p = 0.125; F = 0.047, df = 4, p = 0.06; F = 1.225, df = 4 [illegible] = 0.193) only in field population B, with a decrease of 2.71 %, 4.47%, and 1.88%, respectively. The content of trehalose decreased significantly (F = 0.072, df = 4, p = 0.033; F = 1.323, df = 4, p = 0.015; F = 3.484, df = 4, p = 0.048) in all three populations, with a decrease of 21.3%, 14.5%, and 38.4%, respectively, and the activity of PFK was increased significantly (F = 2.691, df = 4, p = 0.006; F = 9.414, df = 2.020, p = 0.027; F = 7.620, df = 4, p = 0.032) in the three populations, with an increase of 99.1%, 68.5%, and 53.2%, respectively (Fig 2).

**Fig 2. Influence of $LC_{50}$ rotenone on sugar metabolism in different populations of *A. glycines.***

The influence of $LC_{50}$ rotenone on total sugar content (A), trehalose content (B), and PFK

activity (C) in different populations of *A. glycines*. Different stripes in bars represent different treatments. * indicates that there was a significant difference ($p < 0.05$) between the chemical treatment group and control check group for the adults of *A. glycines* of the same population. Bar heights represent the sample mean and error bars are the standard error of the means.

## Influence of rotenone $LC_{50}$ stress on the content of several metabolic substances in *A. glycines* adults of three populations

After being stressed with rotenone at $LC_{50}$, the content of cholesterol increased significantly ($F = 7.651$, $df = 4$, $p = 0.036$; $F = 2.936$, $df = 4$, $p = 0.013$; $F = 1.517$, $df = 4$, $p = 0.037$) in all three populations, with an increase of 39.2%, 69.7%, and 32.7%, respectively. The activity of GST decreased significantly ($F = 2.110$, $df = 4$, $p = 0.032$; $F = 0.036$, $df = 4$, $p = 0.007$; $F = 12.430$, $df = 4$, $p = 0.003$) in all the three populations, with a decrease of 22.6%, 18.9%, and 71.6%, respectively, and the content of CYP450 increased in the three populations, but only increased significantly ($F = 1.123$, $df = 4$, $p = 0.135$; $F = 0.693$, $df = 4$, $p = 0.051$; $F = 0.258$, $df = 4$, $p = 0.006$) in the laboratory population, with an increase of 14.8%, 24.2%, and 76.7%, respectively (Fig 3).

**Fig 3. Influence of $LC_{50}$ rotenone on other metabolic substances in different populations of *A. glycines*.**

The influence of $LC_{50}$ rotenone on the cholesterol content (A), GST activity (B), and CYP450 content (C) in different populations of *A. glycines*. Different stripes in bars represent different treatments. * indicates that there was a significant difference ($p < 0.05$) between the chemical treatment group and control check group for *A. glycines* adults of the same population. Bar

heights represent the sample mean and error bars are the standard error of the means.

## Comparison of the content of main metabolic substances in *A. glycines* adults of three populations before rotenone $LC_{50}$ stress

As shown in Table 2, the content of protein in *A. glycines* adults before the stress was the highest in field population A at 0.534 mg/mL and lowest in the laboratory population, at 0.27 mg/mL; the difference among the three populations was significant (P < 0.05). The activity of protease in population A was the highest, at 733.89 U/mL, and that in the laboratory population was the lowest, at 481.83 U/mL, with a significant difference (P < 0.05) between either field population (A or B) and laboratory population. The content of FAA was the highest in field population A at 659.30 μmol/L and lowest in the laboratory population at 384.82 μmol/L, with a significant difference (P < 0.05) between either field population (A or B) and laboratory population.

The content of total sugar in *A. glycines* was the highest in field population B at 0.368 mg/mL and lowest in the laboratory population at 0.346 mg/mL, with a significant difference (P < 0.05) between either field population (A or B) and laboratory population. The content of trehalose was the highest in population A at 0.29 mg/mL and lowest in the laboratory population at 0.20 mg/mL, with a significant difference (P < 0.05) between either field population (A or B) and laboratory population. The activity of PFK was the highest in field population B at 22.22 U/mL and lowest in the laboratory population at 15.54 U/mL, without significant difference among the three populations.

**Table 2. Content/activity of physiological indexes in *A. glycines* of field population A, field population B, and laboratory population before treatment with $LC_{50}$ rotenone.**

| Physiologically active substance | Insect source | Content/activity |
|---|---|---|
| Protein (mg/mL) | Field population A | 0.[illegible] ± 0.022a |
| | Field population B | 0.464 ± 0.012b |
| | Laboratory population | 0.272 ± 0.012c |
| Protease (U/mL) | Field population A | 733.889 ± 58.680a |
| | Field population B | 626.587 ± 34.104a |
| | Laboratory population | 481.825 ± 6.791b |
| FAA (μmol/L) | Field population A | 659.297 ± 41.329a |
| | Field population B | 620.469 ± 41.352a |
| | Laboratory population | 384.818 ± 17.764b |
| Total sugar (mg/mL) | Field population A | 0.366 ± 0.004a |
| | Field population B | 0.368 ± 0.002a |
| | Laboratory population | 0.346 ± 0.004b |
| Trehalose (mg/mL) | Field population A | 0.293 ± 0.012a |
| | Field population B | 0.280 ± 0.009a |
| | Laboratory population | 0.199 ± 0.027b |
| PFK (U/mL) | Field population A | 16.121 ± 2.779a |
| | Field population B | 22.220 ± 2.587a |

| | | |
|---|---|---|
| | Laboratory population | 16.208 ± 2.328a |
| Cholesterol (mg/mL) | Field population A | 0.042 ± 0.001a |
| | Field population B | 0.041 ± 0.002a |
| | Laboratory population | 0.036 ± 0.002a |
| GST (U/mL) | Field population A | 0.044 ± 0.003a |
| | Field population B | 0.037 ± 0.001a |
| | Laboratory population | 0.035 ± 0.004a |
| CYP450 (ng/mL) | Field population A | 10.612 ± 0.376a |
| | Field population B | 10.184 ± 0.441a |
| | Laboratory population | 7.719 ± 0.908b |

In this table, the content/activity of a substance followed by a different letter indicates a significant difference ($p < 0.05$) between these populations; the data in the table are the mean ± standard error.

The content of cholesterol in *A. glycines* was the highest in field population A at 0.0416 mg/mL and lowest in the laboratory population at 0.0360 mg/mL, without significant difference among the three populations. The activity of GST was the highest in field population A at 0.044 U/mL and lowest in the laboratory population at 0.035 U/mL, with no significant difference among the three populations. The content of CYP450 was the highest in field population A at 10.61 ng/mL and lowest in the laboratory population at 7.72 ng/mL, with a significant difference ($P < 0.05$) between either field population (A or B) and laboratory populations.

# Discussion

The v[illegible]nce test results showed that *A. glycines* individuals from the laboratory population were the most sensitive to rotenone, followed by those from field population A and field population B. The $LC_{50}$ value of individuals from field populations A and B was 1.09 and 1.14 fold higher that of the laboratory population, respectively, indicating that *A. glycines* adults of these field populations had not developed resistance to rotenone. The results of the rotenone stress test showed that the variable quantity of physiologically active substances in the laboratory population changed the most, indicating that the resistance of soybean aphids in laboratory population to the stress of rotenone was the weakest, followed by that of individuals from field population A and field population B, that is, different populations in different cropping patterns had a difference in resistance to rotenone. The analysis of metabolic substances in *A. glycines* adults before being stressed with $LC_{50}$ rotenone in the three populations revealed higher content in the field population than in the laboratory population, indicating that *A. glycines* in field had developed a corresponding adaptability after long-term interspecific competition along with the climatic stress and other factors. The results of this study showed that *A. glycines* in field with different cropping patterns had differing susceptibility to pesticides. This result can provide guidance for the use of precise doses in fields, thus reducing cost and pollution at the same time (Fig 4).

**Fig 4. Stacking diagram of physiological indexes of different populations of *A. glycines*.**

Different stripes in bars represent different populations. The first four bars indicate the increase in content/activity, the next five bars indicate the decrease in content/activity, and the last bar indicates the total change range.

The results showed that after a 24-h stress with $LC_{50}$ rotenone, the activity of protease increased significantly ($p < 0.05$) in *A. glycines* adults in all the three populations, the content of protein decreased significantly ($p < 0.05$), and the content of FAA showed no significant difference. This indicated that protein decomposition was accelerating, most amino acids produced after decomposition were involved in the synthesis of new proteins to maintain normal living activities, and a small part of the amino acids continued to be free in the hemolymph to maintain the balance of blood osmotic pressure of the insect [13, 14, 15, 16, 17]. The trehalose content decreased significantly ($p < 0.05$) in the three populations and the activity of PFK increased significantly ($p < 0.05$); the total sugar content decreased in all the three populations but the decrease was significant ($p < 0.05$) only in field population B, implying the conversion of other substances into sugar, while the glycolysis rate was enhanced to maintain the stability of total sugar content in *A. glycines* [18, 19, 20]. Protein metabolism and sugar metabolism played an important role in the resistance of *A. glycines* to the 24-h stress of rotenone. This result is consistent with Cheng Weixia’s study results on the preference of chosen polysaccharides and soluble proteins as metabolites when *Liposcelis entomo*[illegible]*la* and *Liposcelis bostrychophila* were resistant to poor environments [21].

The results showed that after a 24-h stress with rotenone at $LC_{50}$, the cholesterol content increased significantly ($p < 0.05$) in *A. glycines* adults in the three populations. Simultaneously, *A. glycines* adults’ feeding capability was weakened and the possibility of receiving more cholesterol from food was low. Therefore, the most likely reason is that transportation in *A. glycines* was blocked, which ultimately affected the growth and development of *A. glycines* [22,

23, 24, 25]. The activity of GST was weakened significantly ($p < 0.05$) in the three populations, whereas the content of CYP450 increased but the increase was significant ($p < 0.05$) only in the laboratory population, indicating that CYP450 in *A. glycines* played a role in the response to rotenone stress, presumably due to the increased expression of this gene and may be related to the development of resistance [26, 27]. Previous studies have shown that the resistance of mosquitoes to insecticides was related to the increase in the expression of CYP450 [28], suggesting that we should pay attention to the changes in this gene when using rotenone in the future. The weakened activity of GST may be caused by increased consumption or by the inhibitory effect of rotenone, but the specific reason for this phenomenon needs further study [29, 30, 31].

In summary, after being stressed with rotenone at $LC_{50}$, *A. glycines* showed some effects on some physiological factors, such as the decrease in protein and trehalose content, and blocking of cholesterol transport, that resulted in their metabolic imbalance, slowed movement, and eventual death. *A. glycines* of different populations from different cropping patterns showed a difference in resistance and adaptability to rotenone, therefore, their controlling methods should be adjusted according to the cropping patterns to achieve the goal of effective pest control and reduced environmental pollution.

# Acknowledgments

This work was supported by Special Fund for Construction of Modern Agricultural Industry Technology System (grant number CARS-04); Heilongjiang Science Foundation Project (grant number C2018011).

# Disclosure

The authors declare no conflicts of interest.

# References

1. Liu J, Zhao KJ. Biological control technology of *Aphis glycines*. Insect Knowledge. 2007; 44: 179-185.

2. Wang ZH. Analysis of the causes of *Aphis glycines* outbreaks in 2004 and suggestions for their control. Soybean Bulletin. 2005; 9.

3. Pan Y, Qin ZG, Xi JH. Establishment of a sensitive virulence baseline for *Aphis glycines* glass tube membrane method. Soybean Science. 2010; 29: 483-485.

4. Wang CR, Deng XC, Yin LJ, Song YH, Zhang DY, Shen HB. Analysis of outbreak factors of *Aphis glycines* in Heilongjiang Province in 2004. Soybean Bulletin. 2005; 19-20.

5. Wang HL, Wang BL, Zhou L. Research on the cotton aphid’s landing and population in different cotton varieties. Agric Res Arid Areas. 2006; 24: 218-221.

6. Si YS, Chen JG, Song XD, Gong XY. Population dynamics of soybean aphids in Heilongjiang Province. Soybean Science. 2017; 36: 614-619.

7. Li XJ, Zheng G, Xu B, Li Y, Yu GW, Xing X, et al. Study on the control of *Aphis glycines* by different soybean cultivation models and natural enemies. J Shenyang Norm Univ, Nat Sci Ed. 2014; 32: 129-134.

8. Yang XH. Preliminary study on soybean and early-maturing potato intercropping to control *Aphis glycines*. Heilongjiang Agric Sci. 2013; 55-57.

9. Han LL, Wang K, Li DP, Zhang WL, Cheng Y, Zhao KJ. Effect of potato-soybean, corn-soybean neighbor cropping on population dynamics of main sucking pests and other pests in soybean fields. Acta Entomol Sin. 2016; 53: 723-730.

10. Liang JL, Zeng Z, Gong HL, Liu XY, Miao J. Rotenone's insecticidal mechanism and its application prospect in termite control. Agric Disaster Res. 2015; 5: 13-14.

11. Nawrot J, Harmatha J, Kostova I, Ognyanov I. Antifeeding activity of rotenone and some derivatives towards selected insect storage pests. Biochem Syst Ecol. 1989; 17: 55-57.

12. Zhang J, Qin XW, Yuan FH, Liu J, Huang J, Zhang RJ. Effects of sublethal doses of nitenpyram on fat, protein, soluble sugar, and free amino acids in the brown planthopper. J Sun Yat-sen Univ, Nat Sci Ed. 2011; 50: 88-93.

13. Alexnat, RK. Protease activities in the midgut of Western corn rootworm (*Diabrotica virgifera LeConte*). J Invertebr Pathol. 2009; 100: 169-174.

14. Hua RX, Hou YM, Shi ZH. Changes in the content of physiologically active substances in the water palm star anise iron after domestication at low temperature. Acta Entomol Sin. 2014; 57: 265-273.

15. Satyavathi VV, Mohamed AA, Kumar S, Mamatha DM, Duvic B. The IMD pathway regulates lysozyme-like proteins (LLPs) in the silk moth, *Antheraea mylitta*. J Invertebr Pathol. 2018; 154: 102-108.

16. Cao XL, Haobo J. Building a platform for predicting functions of serine protease-related proteins in *Drosophila melanogaster* and other insects. Insect Biochem Mol Biol. 2018; 103: 53-69.

17. Zhang H, Wu SY, Wang XQ, Lei ZG. Effects of Beauveria bassiana on hemolymph proteins and free amino acids in adults of Onion flies. China Agric Sci. 2017; 50: 591-598.

18. Becker A, Schlöder P, Steele JE, Wegener, G. The regulation of trehalose metabolism in insects. Birkhäuser Verlag Basel. 1996; 52: 433-439.

19. Qin JM, Luo SD, He SY, Wu J. Characteristics and functions of trehalose and trehalase in insects. J Environ Ent. 2015; 37: 163-169.

20. Yu CH, Lu D, Lin RH, Wang XJ, Jiang H, Zhao F. Trehalose——Insect's blood sugar. Insect Knowledge. 2008; 832-837.

21. Cheng WX, Wang JJ, Chen ZY. Comparison of metabolism of energy substances in *Liposcelis entomophila* and *Liposcelis bostrychophila* under insecticide stress. Zool Res. 2005; 26: 545-550.

22. Gilbert LI, O' Connor JD. Lipid metabolism and transport in arthropods. Florkin M, Scheer BT, editors. In: Chemical Zoology. New York: Academic Press; 1970. pp. 229-253.

23. Behmer ST, Nes WD. Insect Sterol Nutrition and Physiology: A Global Overview. Adv Insect Physiol. 2003; 31: 1-72.

24. Jing XF. Sterols: Essential Nutrients for Insects. Acta Entomol Sin. 2013; 50: 575-582.

25. Zhou J, Li J, Weng Q, Luo YQ. Regulation of ecdysone on insect growth and reproductive processes. Chin J Appl Entomol. 2013; 50: 1413-1418.

26. Tomilova OG, Kryukov VY, Duisembekov BA, Yaroslavtseva ON, Tyurn, MV, Kryukova NA, et al. Immune-physiological aspects of synergy between avermectins and the entomopathogenic fungus *Metarhizium robertsii* in Colorado potato beetle larvae. J Invertebr Pathol. 2016; 140: 8-15.

27. Zhu F, Parthasarathy R, Bai H, Woithe, K, Kaussmann M, Nauen, R, et al. A brain-specific cytochrome P450 responsible for the majority of deltamethrin resistance in the QTC279 strain of *Tribolium castaneum*. Proc Natl Acad Sci. 2010; 107: 8557-8562.

28. Wang Y, Song X, Cheng P, Gong MQ. Research progress on cytochrome p450-mediated resistance to mosquitoes. Chin J Vector Biol Control. 2019; 30: 589-592.

29. Brattsten LB, Holyoke CW, Leeper JR, Raffa KF. Insecticide resistance: challenge to pest management and basic research. Science. 1986; 231: 1255-1260.

30. Liu CL, Lu LX, Xu YL, Yang PC, Cui F. Transcriptome analysis of the three major detoxifying enzyme families of the salivary gland of small brown rice planthoppers. Acta Entomol Sin. 2013; 56: 1509-1515.

31. Zhang N, Liu J, Chen SN, Huang LH, Feng QL, Zheng SC. Expression profiles of glutathione S-transferase superfamily in *Spodoptera litura* tolerated to sublethal doses of chlorpyrifos. Insect Sci. 2016; 23: 675-687.

Click here to access/download;Figure;Fig. 1.tif

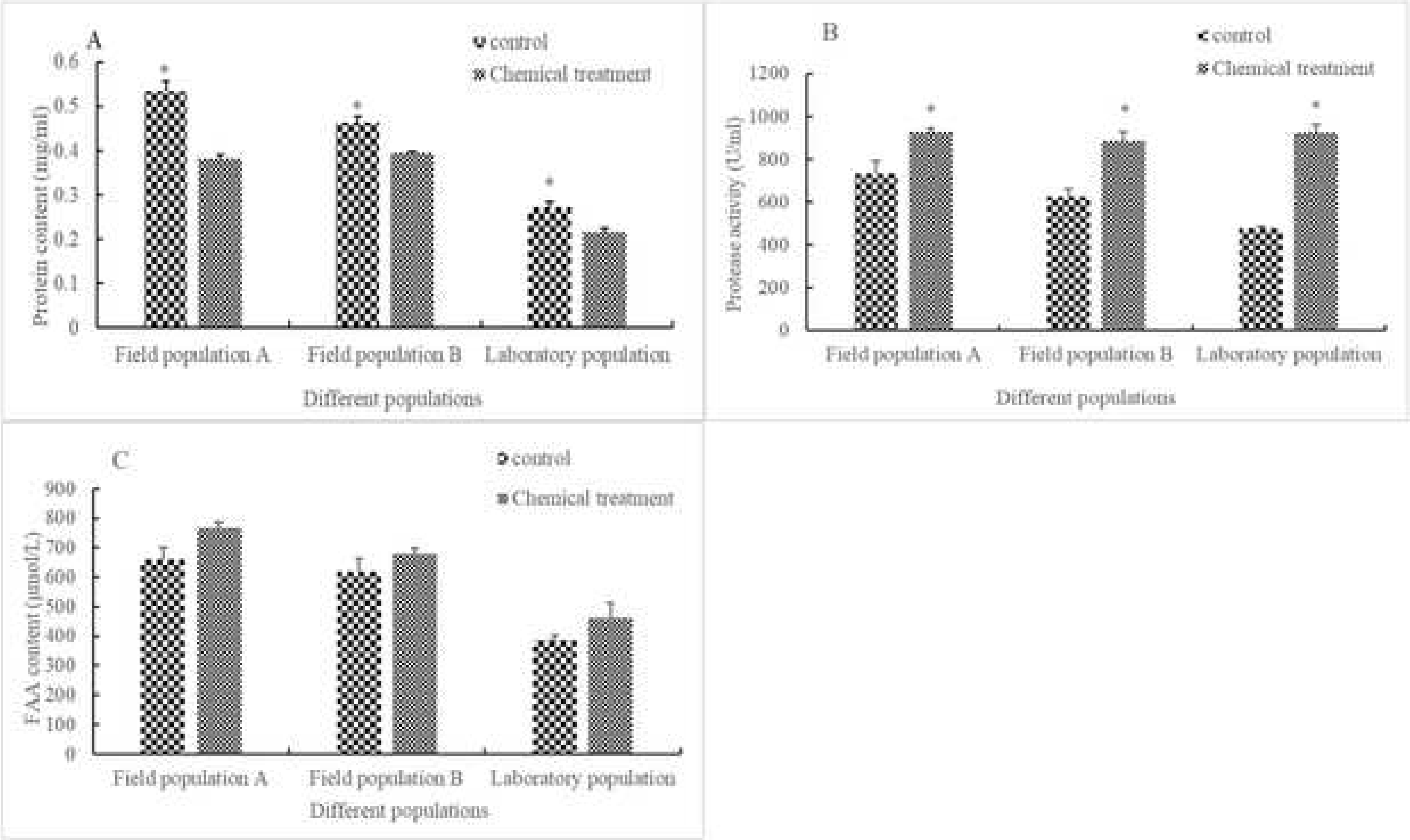

Figure
Click here to access/download;Figure;Fig. 2.tif

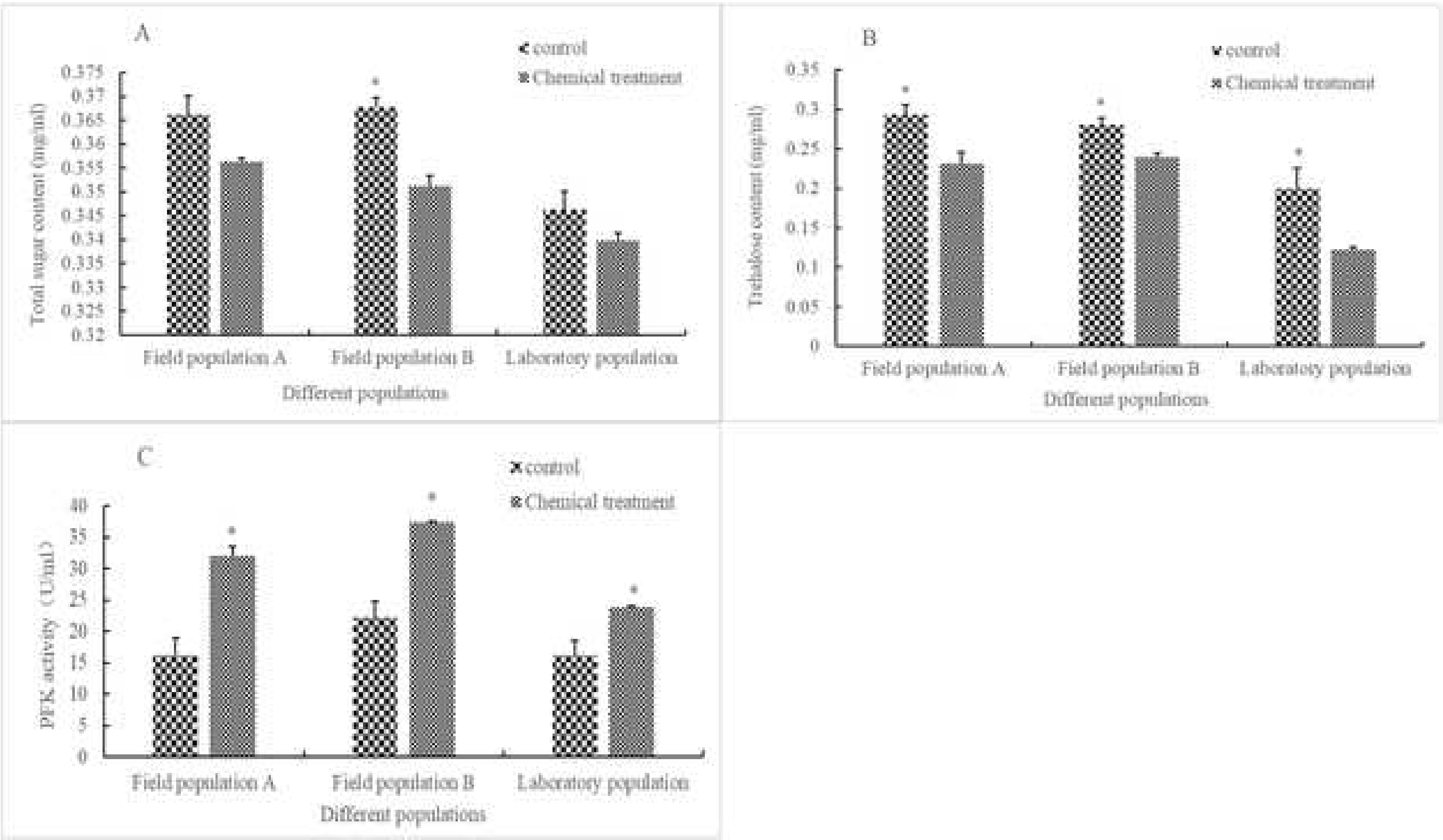

Figure

Click here to access/download;Figure;Fig. 3.tif

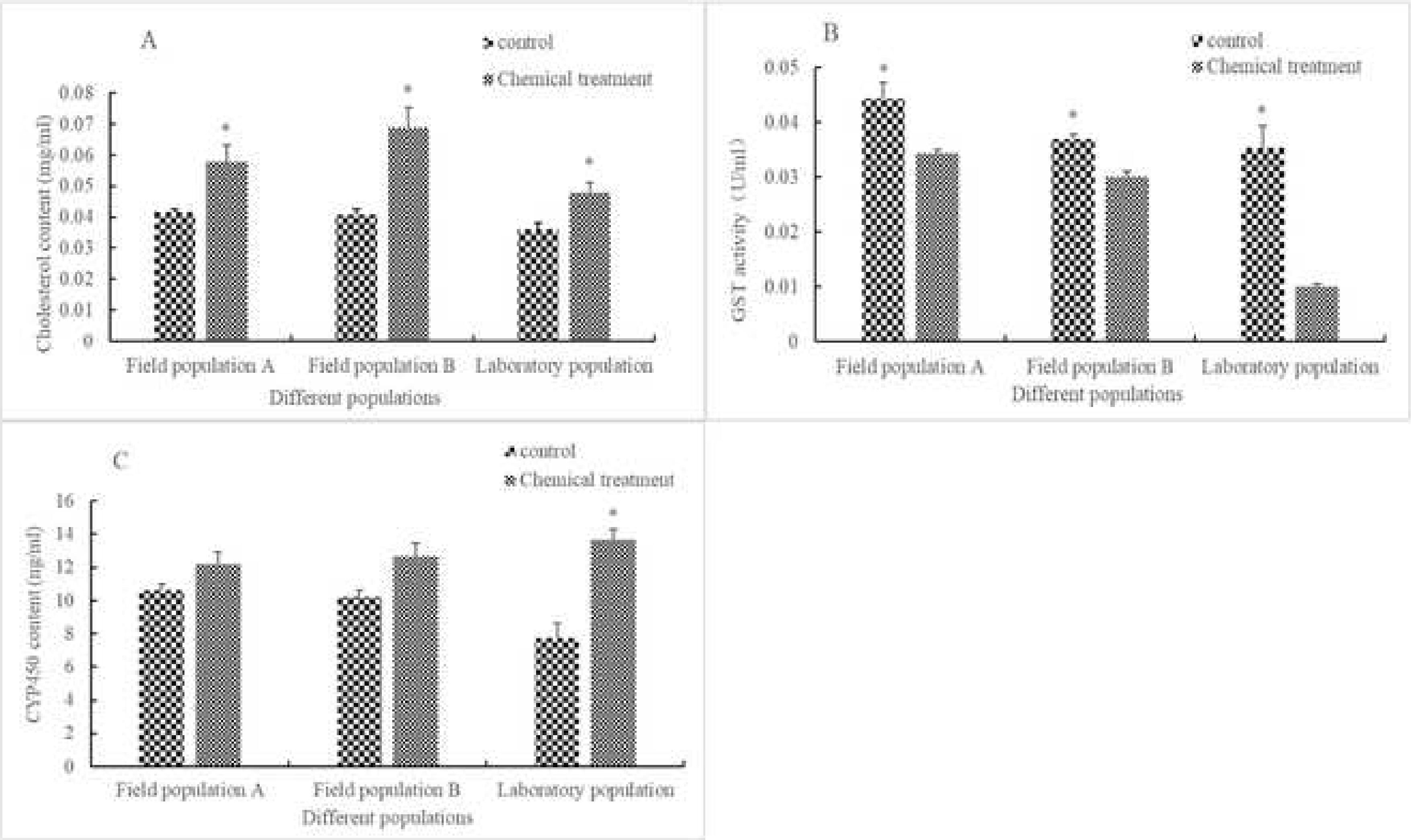

Click here to access/download;Figure;Fig. 4.tif

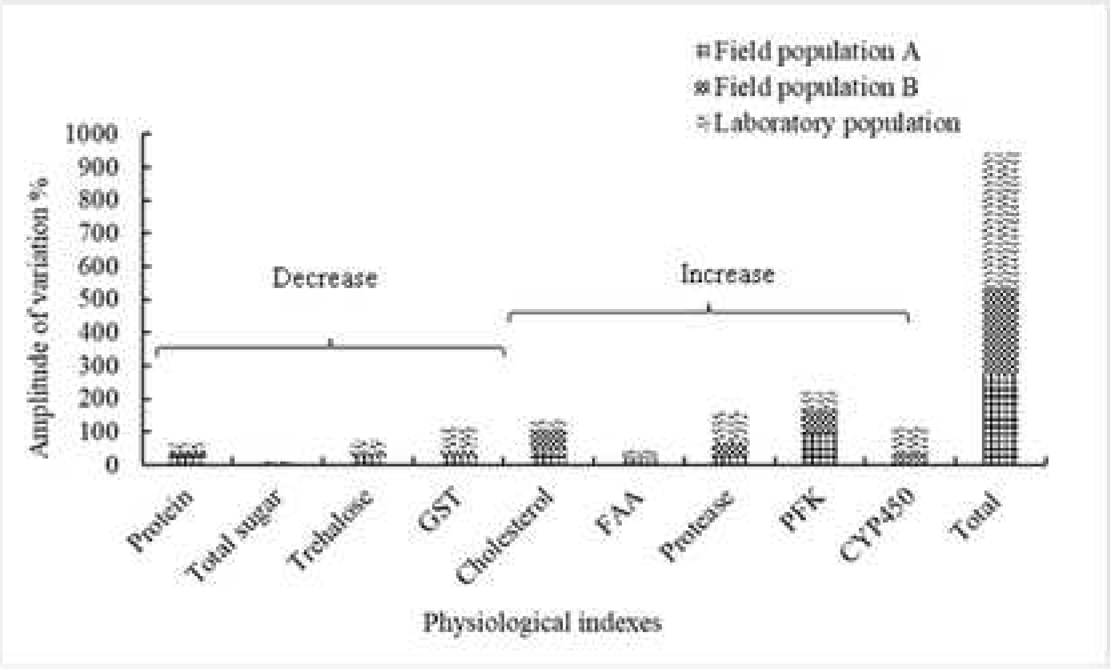

Edited manuscript

Click here to access/download
**Supporting Information**
Edited manuscript_2.doc

Edited manuscript

Click here to access/download
**Supporting Information**
Edited manuscript_ 1.doc
